# Riemannian geometry boosts functional near-infrared spectroscopy-based brain-state classification accuracy

**DOI:** 10.1117/1.NPh.12.4.045002

**Published:** 2025-10-15

**Authors:** Tim Näher, Lisa Bastian, Anna Vorreuther, Pascal Fries, Rainer Goebel, Bettina Sorger

**Affiliations:** a Max Planck Institute for Biological Cybernetics, Tübingen, Germany; b Ernst Strüngmann Institute for Neuroscience in Cooperation with Max Planck Society, Frankfurt, Germany; c Radboud University Nijmegen, Donders Centre for Cognitive Neuroimaging, Nijmegen, The Netherlands; d Meta Reality Labs, Seattle, Washington, United States; e University of Tübingen, Institute of Medical Psychology and Behavioral Neurobiology, Tübingen, Germany; f International Max Planck Research School, Graduate Training Centre of Neuroscience, Tübingen, Germany; g University of Stuttgart, Applied Neurocognitive Systems, Institute of Human Factors and Technology Management (IAT), Stuttgart, Germany; h Maastricht University, Department of Cognitive Neuroscience, Maastricht, The Netherlands

**Keywords:** functional near-infrared spectroscopy, brain-machine interface, Riemannian geometry, machine learning, brain-state classification

## Abstract

**Background:**

Functional near-infrared spectroscopy (fNIRS) has recently gained momentum as a reliable and accurate tool for assessing brain states based on the vascular response to neural activity. This increase in popularity is due to its robustness to movement, non-invasive nature, portability, and user-friendly application. However, compared with other hemodynamic functional brain-imaging methods such as functional magnetic resonance imaging (fMRI), fNIRS is constrained by its limited spatial resolution and coverage with a particularly limited penetration depth. In addition, due to comparatively fewer methodological advancements, the performance of fNIRS-based brain-state classification still lags behind more prevalent methods such as fMRI.

**Methods:**

We introduce a classification approach grounded in Riemannian geometry for the classification of kernel matrices, leveraging the temporal and spatial relationships between channels and the inherent duality of fNIRS signals, specifically oxygenated and deoxygenated hemoglobin. For the Riemannian-geometry-based models, we compared different kernel matrix estimators and two classifiers: Riemannian Support Vector Classifier and Tangent Space Logistic Regression. These were benchmarked against four models employing traditional feature extraction methods. Our approach was tested on seven participants in two brain-state classification scenarios based on the same fNIRS dataset: an eight-choice classification, which includes seven established plus an individually selected imagery task, and a two-choice classification of all possible 28 two-task combinations.

**Results:**

This approach achieved a mean eight-choice classification accuracy of 65%, significantly surpassing the mean accuracy of 42% obtained with traditional methods. In addition, the best-performing model achieved an average accuracy of 96% for two-choice classification across all task combinations, compared with 78% with traditional models.

**Conclusion:**

To our knowledge, we are the first to demonstrate that the proposed Riemannian-geometry-based classification approach is both powerful and viable for fNIRS data, substantially increasing the accuracy in binary and multi-class classification of brain activation patterns.

## 1 Introduction

Functional near-infrared spectroscopy (fNIRS) is an emerging functional neuroimaging technique that has recently gained momentum due to its non-invasive nature, portability, silent data acquisition, robustness against movement artifacts, and favorable trade-off between spatial and temporal resolution.^1^^–^^3^ FNIRS detects hemodynamic responses, which are also measured by functional magnetic resonance imaging (fMRI) and have been linked to changes in neuronal activity.^4^ Both fMRI and fNIRS assess neuronal activity indirectly via the blood-oxygen-level-dependent (BOLD) signal. The BOLD signal is especially linked to neuronal gamma oscillations.^5^^,^^6^ Intriguingly, the BOLD signal reflects changes in both gamma-band activity and neuronal firing rates when they are driven by external stimuli; yet when changes are due to internal state fluctuations, they are captured by BOLD specifically for gamma and much less for firing rates.^7^ This makes gamma oscillations a crucial marker for studying brain activity, particularly in the context of internal state changes.^5^^,^^6^

Unlike fMRI, fNIRS measures two distinct hemoglobin signals: oxygenated (HbO) and deoxygenated (HbR) hemoglobin. Although these signals are naturally anticorrelated,^8^ they are not merely redundant; rather, they can provide complementary information about brain activation.^9^ Despite measuring two separate signals simultaneously, the accuracy of fNIRS-based classification of brain states has yet to reach the level of fMRI. This disparity could partially be attributed to the fact that fMRI benefits from more advanced and well-developed data-analysis techniques due to its more frequent use compared with fNIRS. Consequently, the full potential of fNIRS data analysis has not yet been realized, given its historically less developed and less widely known status. Although fMRI can classify brain states by utilizing spatial differentiation of multivariate activation patterns across the whole brain,^10^ fNIRS is constrained by its limited spatial resolution and coverage, particularly its limited penetration depth. As a result, not all classification methods suited for fMRI data may perform as effectively when applied to fNIRS data.

The current methods for fNIRS-based brain-state classification are often adapted from other functional neuroimaging techniques but are not specifically tailored to the unique properties of fNIRS signals. Traditional classifier approaches include linear discriminant analysis (LDA), support vector classifier (SVC), random forest (RF), and logistic regression (LR), which typically rely on channel-based features such as mean activation, channel variance, regression slope, and zero crossings.^11^^–^^13^ Although more advanced methods, such as convolutional and long-short-term memory (LSTM) neural networks, have been employed to improve classification accuracy, such deep-learning approaches require large amounts of training data, which are often not available.^14^

Given these limitations, there is a need to develop methods specifically tailored to the unique properties of the fNIRS response. In practice, this involves emphasizing the dual nature of fNIRS signals by utilizing the complementary information potentially provided in HbO and HbR data. Advancing data analysis by considering the unique fNIRS-signal properties could bridge the gap between the practicality of fNIRS and the precision seen in fMRI, making fNIRS a more viable alternative for brain-state classification. The aim of this study was to introduce the application of Riemannian-geometry-based models to fNIRS data. To achieve this, we combined HbO and HbR signals in block diagonal channel kernel matrices and subsequently applied Riemannian-geometry-based classification models. Instead of relying on features from individual channels, Riemannian-geometry-based models additionally utilize relationships between channels. To our knowledge, we are the first to demonstrate that the classification of fNIRS data using Riemannian geometry offers a promising alternative to other machine-learning methods. Riemannian geometry machine-learning models work by differentiating brain states based on different spatial co-activations. The framework offers well-established tools that allow for the independent manipulation and therefore tuning of separate signals within the same model. This allows us to effectively capture spatial co-activation patterns separately for both HbO and HbR with only one model. To further optimize task selection for classification applications, we also recorded participant ratings regarding task ease and comfort, as tasks perceived more favorably might inherently provide clearer neural activation patterns. Our approach outperforms traditional methods in both eight- and two-choice classification scenarios. Although Riemannian-geometry-based machine learning is a well-established technique in other classification fields,^15^^–^^21^ we show that it is also a powerful and viable option for fNIRS data.

## 2 Method

### 2.1 Participants

Seven healthy participants (mean age: 32.1±10.6 years, three females) were included in this experiment (Table S1 in the Supplementary Material). All participants were either externally recruited or students and staff members of the Faculty of Psychology and Neuroscience at Maastricht University. Written informed consent was acquired before the experiment. The local ethics committee approved the experiment (Ethics Review Committee Psychology and Neuroscience). Upon successful completion of the experiment, participants received monetary compensation.

### 2.2 General Procedure

Before the experiment, participants received instructions (see Table 1) via email, including short but precise mental-task descriptions to train task performance beforehand. Participants were asked to familiarize themselves with this information and devise their “own (mental) paradigm” (OP): a suitable mental task or process that was not included in the provided task set. The OP task should be familiar to them, and they should feel comfortable executing it upon being cued. We included the OP task to probe whether a participant-defined task would be more effective compared to predefined tasks as it may enhance motivation and task engagement, and thereby imagery success. During the fNIRS session, each participant was seated in a darkened and sound-attenuated room. The fNIRS cap was placed on the participant’s head, and all optodes were adjusted to ensure orthogonality to the scalp and stable optode-scalp contact. During optode placement, participants were presented with the same stimulation (task cues) used for the experiment to practice mental task performance until they felt comfortable. Participants were also instructed to get into a comfortable position and to relax their jaw and facial muscles during the recording session. The fNIRS session lasted for ∼1.5  h including participant preparation. At the end of the session, participants rated experienced pleasantness and ease levels of all eight mental task performances on a 10-point Likert scale (i.e., 1 = extremely unpleasant/extremely difficult to 10 = extremely pleasant/extremely easy). Ratings were not included after each run to ensure that participants performed comparative evaluations across runs and to maintain a smooth and uninterrupted task flow.

**Table 1:** Mental task instructions.

Mental task (abbreviation)	Visual stimulus	Instructions
Mental calculation (MC)	cal	During mental calculation, please calculate multiplication tables (‘the basics’). Perform calculating the multiples of 7, 8, or 9 up to the decuple (e.g., 7-14-21-28-35-42-49-56-63-70, 8-16-24…). Do that at a comfortable but consistent speed. Try to visually imagine the numbers. This might support your imagination. Start from the beginning if necessary.
Mental drawing (MD)	dra	During mental drawing, please imagine drawing simple geometric figures (such as circles, triangles, cubes, etc.) or small contour drawings (e.g., a butterfly, star, car, tree, boat, or house). Do that with your dominant hand and at a comfortable but consistent speed. Try to imagine using a pen. This might support your imagination. Start from the beginning if necessary.
Mental rotation (MR)	rot	During mental rotation, please imagine either a clock with rotating clock hands or a diver jumping from a tower into the water while he spins around several times. Only imagine the moment he is moving in the air. Thus, in your imagination, he should continuously spin around. Again, try to perform this mental task with a comfortable but consistent speed as long as it is indicated.
Mental singing (MS)	sin	During mental singing, please covertly sing any song you know by heart, e.g., a nursery rhyme. Please sing at a comfortable but consistent speed. Try to hear yourself singing. This might support your imagination. Start with the song from the beginning if necessary.
Mental talking (MT)	tal	During mental talking, please covertly recite a text you know by heart (e.g., the prayer “Our father” or any poem you know). Try to hear yourself talking. This might support your imagination. You might have to start from the beginning in case the text ends before the end of the mental task. Start from the beginning if necessary.
Spatial-navigation imagery (SN)	nav	During spatial-navigation imagery, you should imagine to “go” through your house/apartment and look into the different rooms for a moment (e.g., 2 s). Do that at a comfortable but consistent speed. Try to really imagine vividly the various three-dimensional (3D) scenes. The order of the rooms does not matter. However, try to continuously perform this mental task (thus continuously try to imagine the 3D scene of the particular room). After having looked into all rooms of your house/apartment, immediately start from the beginning and perform the spatial navigation task as long as it is indicated.
Tennis imagery (TI)	ten	Imagine playing tennis against a wall (forehand—backhand—forehand—etc.). Focus on the motor aspect (do not visualize the scene). Keep the playing speed constant and perform the tennis imagery as long as it is indicated.

The table presents an overview of the mental tasks performed by participants in the current fNIRS study, including abbreviations in brackets. The visual stimulus presented during the experimental session was limited to three characters (second column). Detailed instructions on how to perform each task were provided to participants prior to the experimental session.

### 2.3 Experimental Runs

**Fig. 1:**
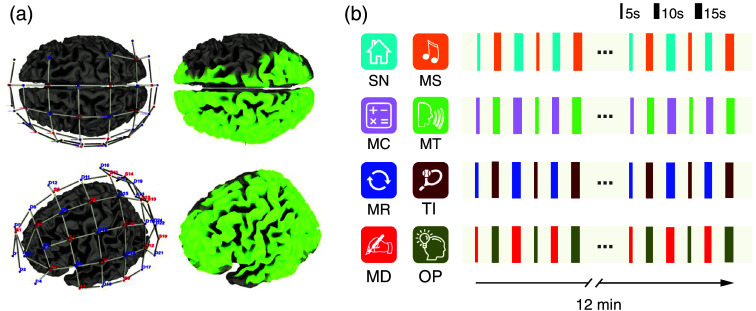
Optode array with resulting NIR light-intensity map and experimental procedure. (a) Optode array (left) and its resulting NIR light-intensity map (right) that indicates the brain coverage (light green regions). (b) Example structure of task pairs used in the four functional runs for one participant. The task pairings varied across participants. Each run started with a 1-min rest period. Task pairs were presented in alternation for 24 trials with 20 s rest periods after each task trial. Encoding durations varied among 5, 10, and 15 s. This pattern was repeated four times in each run and resulted in a total duration of 12 min per run. Abbreviations: MC, mental calculation; MD, mental drawing; MR, mental rotation; MS, mental singing; MT, mental talking; OP, own paradigm; SN, spatial-navigation imagery; TI, tennis imagery.

Participants performed four runs with a total of eight mental tasks [Fig. 1(b)]. Mental tasks included mental talking (MT), spatial-navigation imagery (SN), mental drawing (MD), mental singing (MS), mental calculation (MC), mental rotation (MR), tennis imagery (TI), and the previously devised own paradigm (OP). All tasks except for OP have been shown to elicit differential brain activation patterns using fMRI (unpublished data from the lab).

Participants were instructed to engage in the mental task cued on a computer screen for the duration that the cue was shown. To instruct participants when to engage in mental imagery, three letters of the mental task name were displayed (e.g., “dra” instead of “mental drawing”; see other abbreviations used in Table 1). This was meant to keep visual stimulation similar across tasks and avoid visual stimulation-based differences in cerebral activation. Instructions were presented for 2 s, followed by a fixation cross. Participants were told to engage in the mental task displayed on the screen with the greatest possible consistency. Each trial was followed by a resting period, which was instructed by displaying “res” and during which participants should not engage in a specific thought or task. Each participant performed four mental imagery runs. For each run, two of the eight mental tasks were randomly selected, without replacement, such that across the four runs, each of the eight tasks was selected once. Within each run, the two selected tasks were performed in alternating trials [Fig. 1(b)]. Each trial had a duration that was randomly chosen to be 5 s, 10 s, or 15 s, whereas the duration of the following rest period was always 20 s. Each task was performed for a total of 12 trials per run, resulting in 24 trials per run and 96 trials per participant [Fig. 1(b)]. Across participants, the pairings of tasks were pseudo-randomized to avoid the same pairings across participants.

### 2.4 Data Acquisition

Data were acquired with a NIRScout 16×24 system (NIRx Medizintechnik GmbH, Berlin, Germany) [Fig. 1(a)]. The 24 detectors and 16 sources were positioned according to the international 10-20 electroencephalography (EEG) system. The following list contains the optode positions according to the 10–20 system. Source positions were over AFz, F5, F1, FT7, FC3, FCz, C5, C1, TP7, CP3, CPz, P5, P1, P2, POz, and O1. Detectors were positioned over FPz, FP1, AF3, AF7, AF4, Fz, F3, FC1, FC5, C3, Cz, FC2, T7, CP5, CP1, CP2, P7, P3, Pz, P4, PO7, PO3, PO4, and Oz. Optodes provided full coverage of the left hemisphere and targeted coverage of task-relevant regions in the right hemisphere. This montage was chosen to include areas where we expected to observe mental task-related brain activity. Because most of our mental imagery tasks elicit predominantly left-hemispheric activation including language comprehension and production areas, we sampled large parts of the left hemisphere. We also included bilateral parietal coverage to capture activations associated with mental rotation and spatial navigation, which reliably engage parietal cortices in both hemispheres.^22^^,^^23^ Data were acquired using NIRStar 15.2 (NIRx Medizintechnik GmbH, Berlin, Germany) at 3.47 Hz.

### 2.5 Data Preprocessing

All preprocessing steps were executed using *Satori* (v2.0). According to the optode array [Fig. 1(a)], raw data were preprocessed in 62 channels for oxyhemoglobin (HbO) and 62 channels for deoxyhemoglobin (HbR). Data were trimmed to begin 5 s before the first mental-task trial onset and end 15 s after the last trial offset. The raw time course values were converted to HbO and HbR concentration values using the modified Beer-Lambert law. Subsequent motion correction included spike removal (10 iterations, 5 s lag, 3.5 threshold, 0.5 influence) with monotonic interpolation and Temporal Derivative Distribution Repair (TDDR), preserving high-frequency components.^24^ Due to hardware limitations, we did not obtain time courses from short channels, which is why we were not able to perform short-separation regression to correct for systemic noise such as heartbeat, respiration, and skin-blood flow.^25^^–^^30^ Instead, a global component regression based on principal component analysis (PCA) was performed, removing the first component. Finally, the signals were filtered using a Gaussian low-pass (0.4 Hz) and a Butterworth high-pass (0.01 Hz) and z-scored. For each individual task trial, we cut the data from cue onset to 10 s post-cue. Note that during the recording of the task, participants performed mental imagery for either 5 s, 10 s, or 15 s, followed by a 10 s rest. To keep trials at the same length, we only utilized the first 10 s post-cue for classification analysis. We estimated several features per channel, including mean, variance, and kurtosis. These features were then input into the traditional classifiers. We chose to keep the length of windows the same, regardless of task performance duration, to avoid introducing a bias in covariance estimation due to different amounts of data. This ensured that covariance and kernel estimates were based on comparable amounts of data across all trials, independent of whether the underlying task duration was 5, 10, or 15 s. Hyperparameters related to the classifiers underwent hyperparameter tuning to optimize their performance. For the Riemannian methods, different kernel matrices were estimated from the 62 HbO and HbR channels. The estimation of these kernel methods was according to several different covariance and (non)-linear kernel functions, which were also considered hyperparameters of the Riemannian models. The resulting matrices served as features for the Riemannian classifiers, which also underwent hyperparameter tuning.

### 2.6 Classifiers

We compared 14 classifiers: ten Riemann-based models and four traditional models. The traditional models were LDA, LR, SVC, and RF. We computed standard features (mean, variance, peak, zero-crossings, skew, and kurtosis) for these models as described in the fNIRS literature.^11^ The hyperparameters for each model can be found in the Supplementary Table S2. Hyperparameters were optimized using an exhaustive grid search, in which all possible parameter combinations were evaluated using five-fold cross-validation.

### 2.7 Kernel Matrix Estimation

For EEG-based BCIs, classification based on covariance matrices is standard practice.^15^^,^^31^^,^^32^ These matrices, specifically termed “channel covariance matrices,” are derived from the covariances between channel pairs. Covariance measures the extent to which two time series vary together: a positive covariance indicates that increases in one time series are matched by increases in the other, whereas a negative covariance suggests that an increase in one corresponds to a decrease in the other. However, note that a covariance of zero does not necessarily denote independence between the time series; rather, it indicates that there is no linear relationship between them.

We used several different kernel functions to obtain the kernel matrices between channels, employing the following estimators: the correlation coefficient matrix (“corr”), the numpy-based covariance matrix (“cov”), Huber’s M-estimator based covariance matrix (“hub”), the Ledoit-Wolf shrunk covariance matrix (“lwf”), the oracle approximating shrunk covariance matrix (“oas”), the Schaefer-Strimmer shrunk covariance matrix (“sch”), the scikit-learn empirical sample covariance matrix (“scm”), Tyler’s M-estimator based covariance matrix (“tyl”), and the polynomial kernel (“poly”), which adds nonlinearity by raising the dot product to a specified power, the radial basis function (RBF) kernel (“rbf”), which measures similarity based on the distance between input vectors; the Laplacian kernel (“laplacian”), which is similar to the RBF kernel but uses the L1 norm for distance measurement, and the cosine kernel (“cosine”), which measures the cosine of the angle between input vectors. All kernel functions were implemented using scikit-learn and pyriemann using default parameters.

Let $K_{\mathrm{HbO}}$ be the kernel matrix for the HbO channels, and $K_{\mathrm{HbR}}$ for the HbR channels, where the kernel function κ(·,·) computes the relationship between pairs of channels. Formally, for an input time series $X\in R^{c\times t}$, composed of c channels and t time samples, the elements of the kernel matrices are defined as

$$K_{i,j}^{\mathrm{HbO}}=\kappa (X[\mathrm{HbO}{]}_{i},X[\mathrm{HbO}{]}_{j}),\;K_{i,j}^{\mathrm{HbR}}=\kappa (X[\mathrm{HbR}{]}_{i},X[\mathrm{HbR}{]}_{j}).$$

Here, $X[\mathrm{HbO}{]}_{i}$ and $X[\mathrm{HbO}{]}_{j}$ denote the i’th channel time series for HbO and HbR, respectively. The matrices $K_{\mathrm{HbO}}$ and $K_{\mathrm{HbR}}$ capture the intrinsic channel-to-channel relationships within their chromophore.

Instead of classifying HbO and HbR kernel matrices separately with different classifiers, we can combine them in a block diagonal matrix. A block diagonal matrix is a square matrix composed of smaller square matrices, or “blocks,” along its diagonal and zero entries elsewhere.

The combined kernel matrix, $K_{\text{block}}$, structured as a block diagonal of $K_{\mathrm{Hbo}}$ and $K_{\mathrm{HbR}}$, is defined as:

$$K_{\text{block}}=K_{\mathrm{Hbo}}⊕K_{\mathrm{HbR}}=[\begin{array}{cc} K_{\mathrm{Hbo}} & 0\\ 0 & K_{\mathrm{HbR}} \end{array}].$$

It is important to note that during hyperparameter tuning, we allow κ(·,·) to be (nonlinear) kernel functions as well as covariances with the different estimators, as described above. κ(·,·) can also be different for HbO and HbR. To ensure the numerical stability of the kernel matrices, we additionally regularized $K_{\mathrm{HbO}}$ and $K_{\mathrm{HbR}}$ separately before combining them to $K_{\text{block}}$.

This regularization was done via shrinkage of the matrices, allowing different shrinkage parameters for each chromophore. This approach emphasizes flexibility for each oxygenation type and allows the classifier to be tuned separately to HbO and HbR data while ensuring the SPD property of the matrices. We termed this kernel matrix estimation “super kernel.” A Python implementation of our super kernel estimator can be found in the PyRIEMANN documentation (called HybridBlocks).

By contrast, a full kernel matrix estimation does not permit such tailored optimization, which can limit performance. For full kernel matrix estimation, the approach is essentially the same as described earlier; however, only one kernel function is picked for both HbO and HbR. These full kernel matrices also include the interactions between HbO and HbR channels, whereas these interactions are set to zero for the block diagonal matrices. This means that the full kernel matrices can contain more information than the block diagonal matrices. However, they also lack the ability to tailor kernels separately for HbO and HbR and occupy a higher dimensional manifold.

### 2.8 Co-Activation Comparison and Mean Kernel Matrices

To test whether HbO and HbR show different co-activation patterns, we compared distances between the mean kernel matrices. For this, we first computed the mean kernel matrix for each task and for each participant, separately for HbO and HbR. Note that all mean kernel matrices were estimated with the Riemannian metric.^33^^,^^34^ Next, we computed the pairwise Riemannian distances^35^ between all pairs of tasks (n=28). The resulting distance vectors for HbO and HbR were transformed into discrete probability distributions. Subsequently, we computed the Wasserstein distance between these vectors. We used the Wasserstein distance because it provides a natural metric for comparing distributions based on shape and magnitude, allowing an intuitive interpretation of differences between HbO and HbR kernel matrices. The Wasserstein distance, also known as Earth mover’s distance, can be thought of as the minimum “cost” required to transform one distribution into the other. The “cost” here is calculated based on the amount of probability mass that needs to be moved and the distance it needs to be moved. Intuitively, it represents a difference in shape between the two distributions. The logic is as follows: If HbO and HbR kernel matrices both contain the same information, the distribution of pairwise distances between the individual task pairs is similar. This would lead to a small Wasserstein distance. Likewise, if the two chromophores contain different information and can hence differentiate between task pairs, the distance will be larger. We calculated the Wasserstein distance between HbO and HbR pairwise task distances for all participants and tested whether this distribution differed significantly from zero with a one-sample t test.

### 2.9 Classification Approach

The dataset used in this study consisted of eight different mental tasks with 12 trials each for a total of 96 trials per participant. Given the relatively small sample size, we opted against splitting data into separate training, testing, and validation sets. Instead, we employed a repeated and stratified k-fold cross-validation approach, which is a common approach in machine-learning scenarios with small datasets. Specifically, we employed repeated (n=5) five-fold stratified cross-validation to compute the accuracy score for model comparisons. Although this method is widely accepted and helps mitigate the variability in small datasets, it is important to note that it can sometimes overestimate the accuracy compared with using a separate test set.^36^ However, we performed the same approach for both traditional models and the Riemann models. All computations were performed on an HPC cluster using SLURM, ACME, scikit-learn, and pyriemann.

### 2.10 Relative Improvement Score

We calculated a relative improvement score for Riemann models compared with traditional models for each participant. This score normalizes the improvement by considering the proximity of the baseline accuracy to the maximum possible accuracy (i.e., 1). The relative improvement score is defined as follows:

Let $A_{\text{baseline}}$ be the accuracy of the baseline model, and $A_{\text{new}}$ be the accuracy of the new model. The relative improvement score RIS is calculated using the equation

$$\mathrm{RIS}=\frac{A_{\text{new}}-A_{\text{baseline}}}{1-A_{\text{baseline}}}.$$

This formula scales the observed improvement by the remaining potential for improvement, given the baseline performance. The numerator, $A_{\text{new}}-A_{\text{baseline}}$, represents the absolute improvement in accuracy achieved by the new model. The denominator $1-A_{\text{baseline}}$ represents the maximum possible improvement from the baseline model’s accuracy to perfect accuracy. By using this relative improvement score, we account for the fact that achieving improvements is more challenging as the baseline accuracy approaches 1.

### 2.11 Statistical Tests for Model Comparison

We compared the classification accuracies of traditional methods with the Riemann methods using paired t-tests. Because we used ten Riemann models and four traditional models, we calculated the average accuracy for each participant and each model type using a bootstrap procedure (n=1000). The bootstrapping was applied to the cross-validation accuracies to obtain a more robust estimate of the distribution and to compute 95% confidence intervals. This allowed us to compute the mean of the bootstrapped accuracy distribution and then perform the t tests on these bootstrapped means.

For the best-performing model for the two-choice and eight-choice comparison, we determined whether the repeated stratified five-fold cross-validation accuracy was above chance level. The theoretical chance levels for the two- and eight-choice comparisons are 50% and 12.5%, respectively. However, these numbers are only valid for an infinite number of samples. To better estimate classification performance, we used a permutation-based approach where we shuffled the labels (n=500) and retrained models on the shuffled labels.^37^ All analyses used a significance threshold of P<0.05 unless stated otherwise.

## 3 Results

### 3.1 Leveraging the Inherent Duality of fNIRS Data with Block Diagonal Matrices

**Fig. 2:**
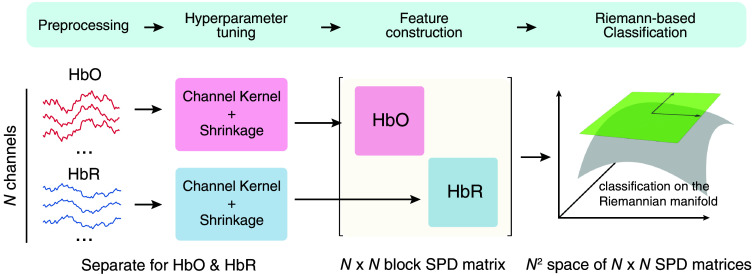
Processing pipeline of Riemannian fNIRS classifiers using block diagonal matrices as features. The data are first preprocessed according to standard pipelines. The subsequent hyperparameter tuning finds the best kernel functions and regularization for HbO and HbR separately. Each combination of hyperparameters is evaluated using a five-fold stratified and repeated cross-validation (five repetitions). The resulting individual kernel matrices are then combined in a block diagonal matrix, which serves as a feature for each trial. Several Riemannian-based classifiers were evaluated for classifying fNIRS data across participants.

To optimally use the information provided by both HbO and HbR, we designed models that use block diagonal matrices for classification. This has the advantage that the manifold on which the data are classified contains information from both HbO and HbR signals that the model can leverage. The pipeline for fitting and estimating these models is illustrated in Fig. 2.

The process begins with standard fNIRS preprocessing steps (detailed in the “Methods” section), during which the data are separated into HbO and HbR signals. After preprocessing, the HbO and HbR data are processed separately (Fig. 2). For each chromophore, we compute a kernel matrix with different estimators according to the hyperparameter set. This separation allows the hyperparameter search to identify the optimal function for each chromophore, optimizing the sensitivity and overall performance of the model. Following the estimation, the HbO and HbR matrices are individually regularized using shrinkage methods. This regularization step is crucial for stabilizing the matrices, especially given the high dimensionality of the data. Once regularized, the two matrices are combined into a block diagonal matrix, which is then used as input for a classifier (Fig. 2).

This method offers a significant advantage by allowing the most efficient kernel and shrinkage parameters to be determined for each signal type individually.

We benchmarked 14 different classifiers in two different settings: classifying eight tasks and classifying two tasks. For each setting, we evaluated four traditional machine-learning models and ten Riemannian-geometry-based models. The traditional models were LDA, LR, RF, and SVC. These models were trained using a traditional feature set comprising channel mean, variance, kurtosis, and skewness.^11^ These features were computed per channel separately for HbO and HbR.

The ten Riemannian models are differentiated by variations in feature estimation and classifiers. We assessed a Riemannian-adapted SVC and LR in the tangent space of the Riemannian manifold. The feature estimation techniques explored were as follows:

a) Full kernel matrix estimation: This approach concatenated HbO and HbR signals into a single 124×124 kernel matrix. As an SPD matrix in $R^{124\times 124}$, its Riemannian manifold has dimension $\frac{124\times 125}{2}=7750$.
b) Block diagonal covariance matrix: We constructed two separate 62×62 covariance matrices for HbO and HbR and placed them on the diagonal, zeroing out cross-terms. This yields a 124×124 block-diagonal SPD matrix whose manifold dimension is the sum of the two 62×62 blocks, i.e., $2\times \frac{62\times 63}{2}=3906$.
c) Block-kernel matrix: Here, the HbO and HbR blocks represented channel kernel functions instead of covariances.
d) Super-kernel block diagonal matrix: In this approach, the HbO and HbR blocks could represent either covariance or kernel matrices.

### 3.2 Kernel‐Based Covariance Analyses Reveal Complementary HbO and HbR Patterns

**Fig. 3:**
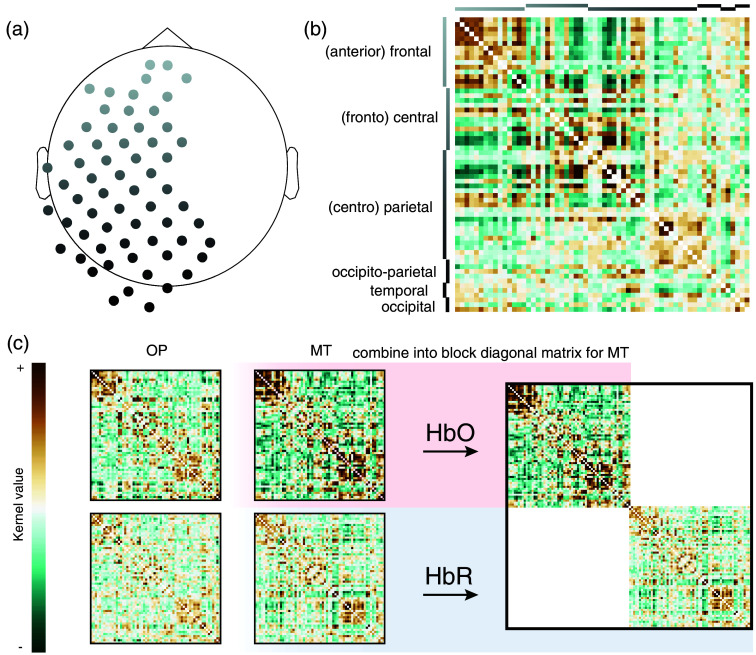
Example average kernel and block diagonal matrices. (a) FNIRS channel map. (b) Example kernel matrix for P01 and task MT. Rows and columns of the matrix are reordered according to the respective EEG locations of the channels. (c) Example average kernel matrices from participant P01 for HbO (top row) and HbR (bottom row) for task OP (left column) and task MT (right column). For task MT, the HbO and HbR kernel matrices are stacked into a block diagonal matrix on the right; the same is done for task OP, but not illustrated here for space reasons. Note that the diagonal (channel variances) is set to zero for illustration purposes. MT, mental talking; OP, own paradigm.

Only a few studies have utilized channel relationships, such as kernel functions, to classify fNIRS data. However, those who have explored this approach demonstrated that kernels outperformed traditional fNIRS features for classifying mental tasks.^38^ For kernel matrices to be an effective feature for classification, it is crucial to record the activity from different brain regions as cleanly separated signals (minimizing mixture) and to obtain as many independent brain-activation measurements as possible. This can be optimally achieved by covering a large cortical area. Thus, our montage included 62 optode channels [Fig 3(a)], spanning the whole left hemisphere from occipital to frontal regions as well as parts of the right hemisphere. We sorted the individual channels of the kernel matrices according to their locations in the 10 to 20 EEG system in (anterior)-frontal, (fronto)-central, (centro)-parietal, occipital-parietal, temporal, and occipital. Figure 3(a) shows this sorting for two example kernel matrices (HbO and HbR) and the tasks “mental talking” and “own paradigm” (all eight tasks for this participant can be found in Fig. S2 in the Supplementary Material). Generally, for the group, kernel values were higher for HbO than HbR (t=5.08, $P=2.26\times 10^{-3}$), likely due to the different signal-to-noise ratios of the two signals. Although scaling covariance matrices affects the eigenvalues, it does not affect the eigenvectors.

We tested whether HbO and HbR displayed different co-activation patterns by comparing the distributions of pairwise distances between all possible combinations of two tasks. We found that the average Wasserstein distance was significantly greater than zero, indicating that HbO and HbR pairwise distances contain different information structures (t=4.68, P=0.003). Generally, HbO showed a higher average distance between task pairs compared with HbR (t=3.25, P=0.017).

The duality of fNIRS data suggests that one could use HbO and HbR as separate features for classification. However, adding more features to classification analyses does not necessarily improve classification accuracy. Riemannian geometry provides an elegant framework for combining different kernel matrices (features) into a single matrix by stacking them along the diagonal, resulting in a block diagonal matrix [Fig. 3(c)]. Thus, Riemannian geometry is particularly well-suited to manage the duality inherent in fNIRS data.

### 3.3 Riemannian Models Consistently Outperform Other Models

**Fig. 4:**
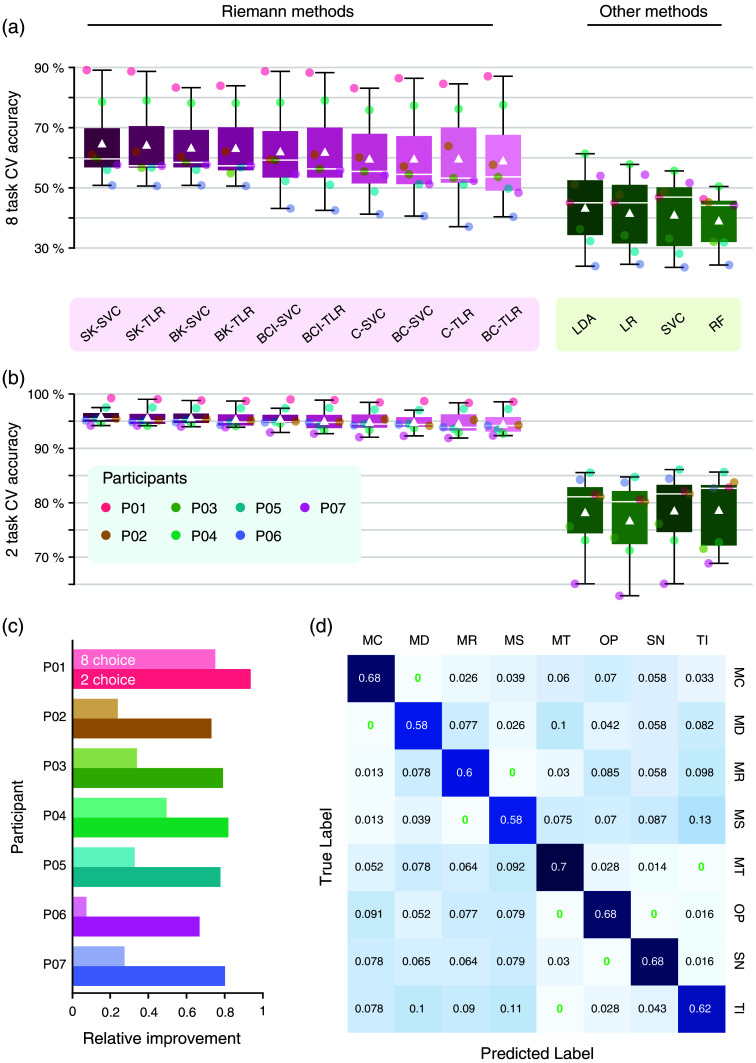
Classification results. Classification results of ten different Riemann-based methods (pink) and four traditional methods (green). (a) The mean accuracy for the one multi-class classification of all combinations of eight tasks. The box plot shows the median for each model as a white bar and the mean as a white triangle. Outliers are depicted as circles. (b) Same as panel (a), but for the 28 binary classifications of all combinations of two tasks (n=7 participants in both cases). (c) Relative improvements of average accuracies from traditional to Riemann methods per participant. Lighter colors indicate relative improvements for the eight-choice paradigm, and darker colors indicate relative improvements for all possible two-choice combinations. (d) Mean confusion matrix for the eight-choice comparison, using the best-performing model (US_SVC). Cells for tasks that were never confused with other tasks are marked in green. Abbreviations: SK, super-kernel; BK, block-kernel; C, covariance; BCI, block covariance individual; SVC, (Riemannian) support vector classifier; TLR, space logistic regression; RF, random forest; LDA, linear discriminant analysis; LR, logistic regression.

As the final step of our model fitting process, we classify the previously estimated kernel matrices using either Riemannian-adapted classifiers or traditional machine-learning models. Because our dataset includes eight distinct mental-task conditions, it provides an ideal opportunity to test the potential of co-activation-type classification in distinguishing among multiple tasks. For this eight-choice classification, the traditional models (LDA, LR, RF, and SVC) yielded a mean classification accuracy of 42% (SD = 0.120, 95% CI = [0.303, 0.525]) (all P<0.01, permutation tests). By contrast, Riemannian models significantly outperformed traditional models, achieving a mean accuracy of 62% (SD = 0.148, 95% CI = [0.482, 0.755]) [bootstrapped paired t test, t=4.81, $P=2.98\times 10^{-3}$, d=1.817; see Fig. 4(a)].

In addition to evaluating the eight-choice classification performance across all tasks, we also explored a binary classification scenario by examining all possible pairwise combinations of the eight tasks, resulting in 28 different binary comparisons. Here, the traditional models achieved a mean accuracy of 78% (SD=0.073, 95% CI = [0.711, 0.846]) with all classification accuracies above chance level (all P<0.01, permutation tests). The Riemannian models outperformed the traditional models, achieving an average classification accuracy of 95% (SD = 0.020, 95% CI = [0.934, 0.972]) [bootstrapped paired t test, t=7.65, $P=2.50\times 10^{-4}$, d=2.895; see Fig. 4(b)].

The “super-kernel SVC” model performed best in both the eight-choice and two-choice comparisons (65% (SD = 0.138, 95% CI = [0.519, 0.775]) and 96% (SD = 0.112, [0.944, 0.975]) accuracy, respectively). Overall, models utilizing block diagonal matrices (n=8) as features outperformed those based on full-kernel matrices (models: C-SVC, C-TLR) (n= 2) for both two-choice comparison (t=7.57, $P=2.77\times 10^{-4}$, mean difference = 0.075, SD = 0.026, 95% CI = [0.051, 0.099], d=2.86) and eight-choice comparison (t=6.34, $P=7.23\times 10^{-4}$, mean difference = 0.104, SD = 0.043, 95% CI = [0.064, 0.144], d=2.39).

To quantify the improvement, we calculated a relative improvement score comparing the performance of traditional models to Riemannian models. This analysis revealed that the improvement was more pronounced in the two-choice comparisons than in the eight-choice comparisons for all participants [t=−8.37, $P=1.58\times 10^{-4}$, mean difference=−0.431, SD = 0.136, 95% CI=[−0.557,−0.305], d=−3.16, see Fig. 4(c)].

Using our best-performing model, we analyzed the average confusion matrix for the eight-choice classification [Fig. 4(d)]. The results revealed that “mental talking” (MT) had the highest accuracy in predicted versus true labels, with 70% correctly classified. This was followed closely by “mental calculation” (68%), “own paradigm” (68%), “spatial-navigation imagery” (68%), “tennis imagery” (62%), “mental singing” (58%), and “mental drawing” (58%). Interestingly, certain tasks showed complete separability; for example, “mental talking,” the most distinguishable task, was never mistaken for “own paradigm” or “tennis imagery.” Similarly, “mental calculation” was never confused with “mental drawing,” “mental rotation” was never confused with “mental signing”, and “own paradigm” was never confused with “spatial-navigation imagery.” These results suggest that certain task pairs may generate more orthogonal co-activation patterns than others, which can be effectively captured by our proposed methods. However, the classification accuracy for each task did not show a significant correlation with participants’ ratings of ease and pleasantness (all P>0.05, Spearman rank correlation; see Fig. S1 in the Supplementary Material).

## 4 Discussion

In this study, we demonstrate that classification models based on Riemannian geometry offer a powerful and robust method for classifying brain states with fNIRS. We demonstrate that Riemannian models consistently outperform traditional machine-learning models in both binary and multi-class classification scenarios (illustrated in Fig. 4). In addition, our study underscores the benefit of leveraging the complementary information inherent to HbO and HbR signals captured by fNIRS. Stacking HbO and HbR signals in block diagonal matrices, as opposed to full covariance matrices, significantly enhances classification performance. Specifically, the combination of a block diagonal matrix as a feature together with a Riemannian SVC achieved the highest accuracy among all models in both the eight-choice and two-choice classification (see Fig. 4), with an average of 65% and 96% cross-validation accuracy, respectively.

### 4.1 Benefits of Combining Riemannian Geometry With a Block Diagonal Approach

Riemannian models consistently outperformed traditional models such as LR, SVC, LDA, and RF (see Fig. 4). This difference in performance likely stems from the distinct features each approach utilizes for classification. Although traditional models rely on statistical features computed from individual channels,^13^^,^^39^ Riemannian models harness information from channel variances and interactions.^15^ Because brain processes occur within interconnected networks, capturing both linear and nonlinear channel interactions can substantially improve classification accuracy.^40^ Unlike other statistical features such as mean, kurtosis, or zero-crossings, kernel matrices, which serve as features in Riemannian approaches, can capture these interactions. For these kernel matrices, nonlinear channel kernels can offer an improvement over regular covariances.^41^

Among the Riemannian models, those employing block diagonal matrices as features outperformed models using full kernel matrices at the group level (see Fig. 4). The block diagonal approach allows for the concatenation of multiple SPD matrices, each estimated using potentially different kernel functions and regularization strengths. These models have a larger hyperparameter set, and this additional complexity allows these models to be more finely tailored to the specific characteristics of the two chromophores. The increased flexibility in hyperparameter tuning likely contributed to the superior performance compared to full kernel models. Given that in our data there appears to be minimal meaningful interaction between the HbO and HbR signals, estimating a full kernel matrix may unnecessarily inflate manifold dimensionality without substantially improving classification performance. By setting the interactions between HbO and HbR to zero, block diagonal matrices eliminate these redundancies, resulting in a manifold with lower dimensionality. This submanifold, corresponding to the block diagonal matrix, is therefore more compact and likely better suited to capture the essential structure of the data. The primary advantage of block diagonal matrices lies in independently optimizing kernel selection and regularization parameters for HbO and HbR, maximizing their distinct informational contributions.

The best-performing model in our study was a super kernel Riemannian SVC (SK-SVC, see Fig. 4). As a powerful linear classifier, the SVC is known for its robust generalization capabilities. When data are not linearly separable in their native space, SVC can map them to a higher-dimensional space where linear separation becomes possible, making it highly efficient in capturing potential nonlinear interactions. Although SVCs traditionally operate with Euclidean feature vectors, they can be extended to incorporate a Riemannian kernel, allowing for kernel matrices to be used as input features.^26^ In this study, we compared a regular SVC with traditional features with the Riemannian SVC with (block) kernel matrices as features (see Fig. 4). The Riemannian SVC, based on the block diagonal approach with separate HbO and HbR tuning, stands out as the most effective method for classifying fNIRS mental imagery data in our study.

### 4.2 Integration of Riemannian Geometry in fNIRS-based Clinical Applications

A rapidly growing field of fNIRS applications is brain-computer interfacing for motor-independent communication and control, where voluntarily generated brain activations/signals are used to encode computer commands. In these systems, the ability to accurately classify distinct patterns of brain activity is crucial for enabling effective communication and control. As fNIRS-based brain-computer interfaces (BCIs) continue to evolve,^42^ integrating advanced analytical techniques such as Riemannian geometry has the potential to significantly enhance the precision and reliability of these systems. Similarly, Riemannian geometry has successfully been used to advance EEG-based BCIs^32^ and extending this approach to fNIRS represents a natural progression. Using mental-imagery tasks, crucial for BCI applications, we show that applying Riemannian geometry to fNIRS data has great potential for the BCI field. Most notable is the substantial increase in classification accuracy achievable using Riemannian models compared with traditional classifiers [see Fig. 4(a)]. In addition, Riemannian models are computationally efficient and typically require less training data compared to deep-learning models. Finally, tools such as the Pyriemann package simplify the implementation of Riemannian models, making them accessible to researchers across various disciplines.

In addition to the general practical benefits of Riemannian models, the block-diagonal approach suggested in this work specifically adds value to real-world fNIRS-based applications. A key advantage of the block‐diagonal Riemannian framework in BCI and clinical contexts lies in its ability to allow for different kernel estimations and regularizations for HbO and HbR covariance patterns while substantially reducing model dimensionality relative to full covariance approaches. This reduction in dimensionality in turn can accelerate classifier training and lower decoding latency. Thus, the block‐diagonal Riemannian approach offers a balanced trade‐off between representational richness and computational efficiency, strengthening its translational relevance for clinical BCI implementations. These advantages could make fNIRS, with its unique HbO and HbR signals, a viable alternative to other BCI input modalities, such as fMRI and EEG.

Currently, EEG remains the predominant method in real-world BCI applications,^43^ with several established classification methods based on Riemannian geometry.^16^^,^^44^^–^^47^ Our study demonstrates that Riemannian geometry may also be well-suited for fNIRS-based BCIs. Thus, the combination of EEG and fNIRS in hybrid BCIs represents a promising future direction.^48^ Future studies could identify co-activation patterns between these two modalities, potentially enhancing BCI performance by leveraging the complementary information from both techniques within a unified Riemannian-geometry framework. The integration of EEG with fNIRS might proceed according to the integration of HbO and HbR exemplified here, namely by means of block diagonal matrices. This integration could further significantly advance BCI technology, offering more robust and versatile systems in which the unique advantages of fNIRS and EEG can optimally be exploited.

For practical BCI applications, it may be advantageous to focus on a smaller subset of tasks, selecting those that are most orthogonal to each other to enhance classification accuracy. Our dataset, which includes eight mental tasks, offers a unique opportunity to identify which tasks are most distinguishable using Riemannian classification (Fig. 4). We identified “mental talking” as the most easily distinguishable task using our analysis approach, likely due to its intuitive nature and close relation to natural thought processes. Interestingly, participant ratings supported this, with “mental talking” ranking second highest for “easiness” and third for “pleasantness” (see Fig. S1 in the Supplementary Material).

Next to the mentioned BCI applications, fNIRS-based classifications using Riemannian geometry could also have significant implications for other clinical applications, considering its accuracy, computational efficiency, and accessibility. As an example, ongoing research in our lab applies the proposed method to further advance earlier attempts for the brain-based diagnosis of remaining awareness in patients with disorders of consciousness.^49^ In these clinical populations, the hemodynamic response often deviates significantly from typical patterns, potentially differing substantially between HbO and HbR signals. For example, in mental imagery, the signal time course greatly depends on how an individual subject performs the task—whether they begin and end immediately or act with a delay. This highlights why our approach, especially extensive hyperparameter tuning and the use of block-diagonal matrices, is particularly suitable for BCIs and other clinical applications.

### 4.3 Limitations and Future Directions

Despite the promising results, several limitations must be considered. The study involved only seven participants who were students and staff members at Maastricht University, which limits the generalizability of the findings. Future efforts to assess the generalizability of our findings should include a larger sample and stratify across demographic variables (e.g., age and sex) and physical characteristics (e.g., hair density and type, and skin pigmentation) and include these variables in the analysis to assess their selective effects on our classification approach.^50^ In addition, the study obtained only 96 trials per participant. This modest amount of data has likely resulted in a relatively low sensitivity. This has however not prevented clear and significant results, supporting the possibility of using this approach in clinical practice with typically restricted recording conditions. Nevertheless, future studies with more data per participant might investigate this aspect in more detail. However, clinical research, particularly in BCI applications, often prioritizes individualized analyses, reducing the necessity for broader population generalization. Note also that despite the limited sample size and the correspondingly low sensitivity, our findings consistently demonstrated that Riemannian models significantly outperformed traditional models at the group level.

However, the observed “complementarity” between HbO and HbR signals that potentially contributed to the superior performance of Riemannian models should be interpreted with caution. We have observed that HbO and HbR signals yield significantly different Riemannian distance distributions, which suggests that each chromophore contributes distinct information to our classifiers. Yet, the canonical hemodynamic response inherently links HbO and HbR in an inverse fashion, and without short‐separation channel regression, extracerebral factors (e.g., scalp blood flow, cardiac and respiratory oscillations) may differentially influence each measure. Consequently, the enhanced decoding accuracy obtained by integrating both HbO and HbR within a block‐diagonal Riemannian framework likely reflects a combination of true cortical dynamics and divergent artifact sensitivities, rather than two wholly independent neural signals. Future work employing short‐distance channels, advanced physiological regression methods, and direct neural validation (e.g., simultaneous EEG) will be critical for disentangling cortical contributions from systemic noise and for confirming the neuronal underpinnings of any observed HbO/HbR divergences.

Although the present study was principally designed to assess the feasibility of applying Riemannian-geometry-based classifiers to fNIRS data rather than to perform a direct comparison of individual mental-task paradigms, our optode montage with a left-hemispheric focus may have conferred uneven signal-to-noise advantages across tasks. Tasks that elicit bilateral activation (e.g., aspects of spatial navigation or complex imagery) might have been underrepresented in our decoding performance metrics. Although classification accuracies were highest for these conditions, we acknowledge that task-specific optode arrangements or fully bilateral layouts might further enhance decoding robustness. Future investigations should therefore explore tailored or bilateral sampling schemes to maximize sensitivity for each cognitive paradigm, thereby refining both the generalizability and the task-specific efficacy of Riemannian classifiers in fNIRS.

Another limitation is the absence of a dedicated train-test split, which might lead to an overestimation of classification accuracy. Rather, we use cross-validation accuracy, which can inflate real-world performance. This could in principle be mitigated by a train-test split of the available data, which was however not feasible due to the small number of trials per subject. Even though cross-validation does not provide an absolute measure of generalization, it allowed for a fair comparison between models, as the same validation protocol and data partitions were applied uniformly across all approaches. Future work may investigate dimensionality reduction strategies (e.g., Riemannian tangent space mapping with feature selection or regularization techniques) and incorporate larger datasets to enable dedicated train-test splits.

In addition, Riemannian models required more extensive hyperparameter tuning than traditional models. Yet, this extended tuning process allowed us to optimize model performance and reach superior performance. Enhanced model sensitivity enables the detection of subtle neural effects, making this approach valuable for advancing our understanding of brain function. Although accuracy is similarly important for BCI applications, time is often a limiting factor in applied fNIRS-BCI settings. BCIs using fNIRS generally work on longer time scales due to the characteristic time delay of the hemodynamic response. In addition, conducting a large hyperparameter tuning might lead to long fitting times. Thus, it may be necessary to reduce the parameter space in BCI and other clinical contexts to achieve an optimal balance between model-fitting time and accuracy. Alternatively, a Riemannian model with a single kernel matrix and a subset of channels could offer a solution that requires less powerful hardware while still surpassing traditional approaches. Future research might investigate the integration of Riemannian geometry with deep-learning techniques to further improve classification performance, a strategy that has shown promise in EEG.^51^

Furthermore, it should be noted that we did not perform a short-distance channel correction to denoise the fNIRS data, which is currently considered best practice. However, at the time of data collection, our fNIRS Scout 16×24 system did not support short-distance channels, which is why we were unable to perform this correction. To mitigate global physiological interference, we instead applied a PCA‐based global component regression. This PCA approach is a viable alternative, when short-distance channel measurements are unavailable, to remove heartbeat, respiratory noise, and skin-blood flow.^52^^–^^54^ Nonetheless, our classifiers achieved robust performance across tasks, indicating that task‐relevant hemodynamic signals were preserved despite residual systemic noise. Future studies should incorporate short‐distance channels to more precisely disentangle cortical from extracerebral contributions.

Due to the short trial durations, the data per trial are also relatively short. We computed covariance matrices over fixed-duration windows to maintain consistency across trials and conditions. Longer recordings per trial would indeed provide more stable covariance estimates and potentially improve stationarity assumptions, but this would come at the cost of increased trial durations and participant fatigue. Our choice reflects a balance between signal reliability and experimental feasibility.

In the current study, “mental talking” was the most distinguishable task out of all eight tasks. Of note is that mental talking may have elicited unconscious movements of the tongue or yawning. These subliminal movements could have contributed to the classification by increasing motor cortical activation. However, previous studies have used “mental talking” successfully for fNIRS-based classification in a movement-disabled patient,^55^ further supporting the distinguishability of the task. Future studies may measure electromyography to detect potentially confounding muscle activity. Note that any such subliminal movements would have been equally available to all classification methods and cannot explain the observed differences between methods.

To validate the utility of a Riemannian-geometry-based approach, the models employed in our rather controlled setting should be tested across diverse settings, from basic research to applied contexts, to determine which models are most effective in various scenarios. Our fNIRS recordings were acquired in a darkened, sound-attenuated laboratory setting chosen to minimize light fluctuations, acoustic distractions, and motion-induced artifacts. By reducing these sources of physiological and optical noise, we were able to obtain a clear benchmark of decoding performance and rigorously compare Riemannian-geometry classifiers under standardized conditions. However, typical BCI deployments (i.e., clinical wards, rehabilitation centers, or home environments) naturally contain environmentally induced noise, which can degrade signal quality. Therefore, future work should extend our findings by systematically evaluating classifier robustness in more ecologically valid contexts. Such studies should investigate adaptive preprocessing strategies (e.g., real-time motion correction, dynamic light compensation), optimize optode layouts for mobile applications, and retrain models on data collected *in situ* to ensure reliable performance outside the laboratory.

## Supplementary Material

10.1117/1.NPh.12.4.045002.s01

## Data Availability

An implementation of the associated code is included in the open-source package *pyriemann*: https://pyriemann.readthedocs.io/en/latest/.
