# Supplementary material for: Riemannian geometry boosts functional near-infrared spectroscopy-based brain-state classification accuracy
Source: Neurophotonics. 2025 Oct 15;12(4):045002. doi: 10.1117/1.NPh.12.4.045002 (PMC12523035; doi:10.1117/1.NPh.12.4.045002)
Supplement: Supplementary file 1 [file NPh_012_045002_SD001.pdf]

Supplementary materials

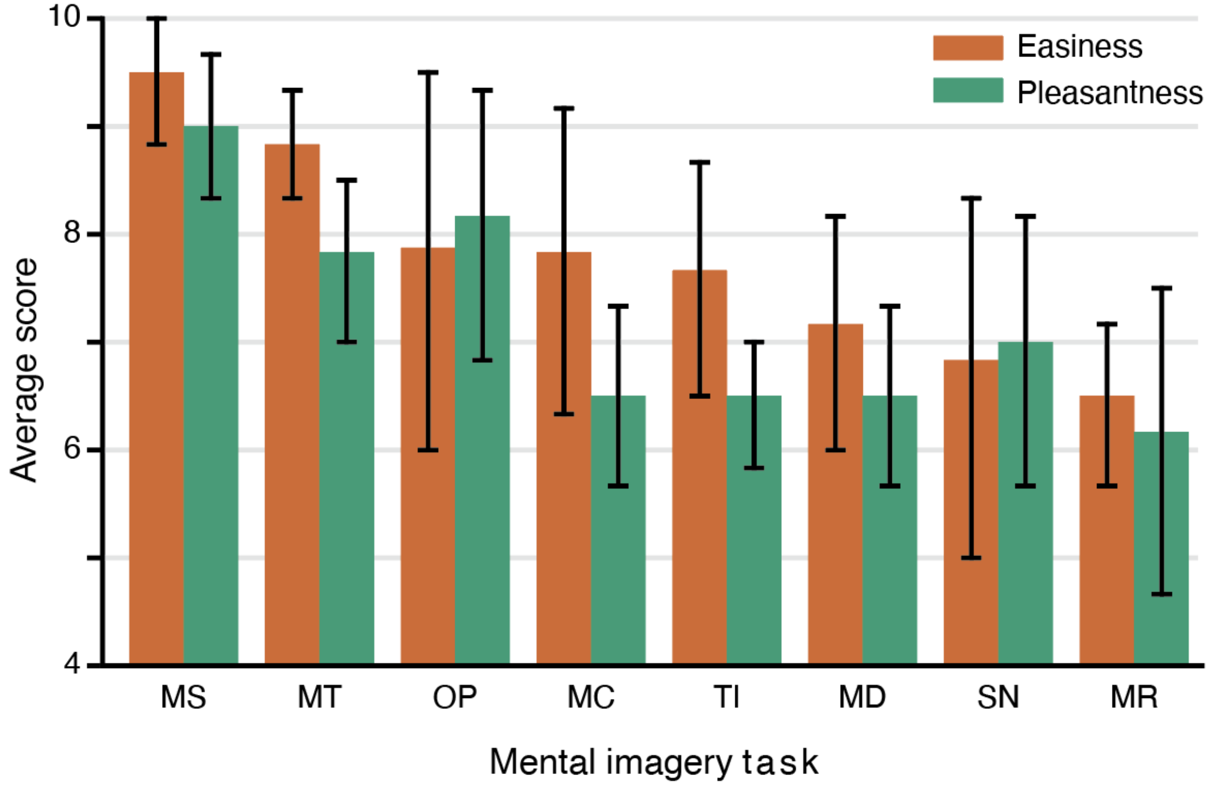

Supplementary Fig S1: Mental task easiness and pleasantness ratings.

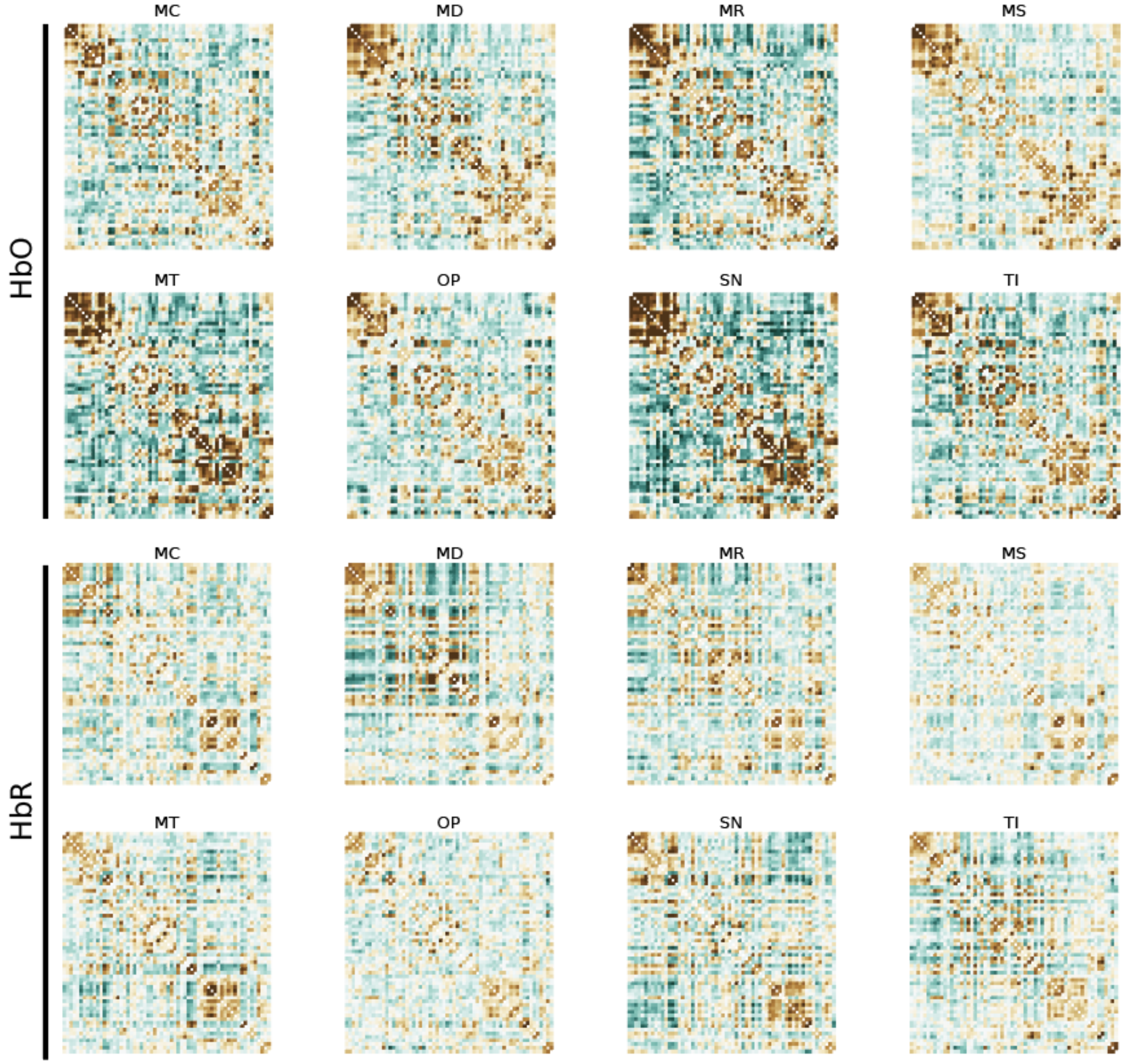

Supplementary Fig S2: Example covariance matrices.

Supplementary Table 1. Participant Characteristics.

Participant	Sex	Age	Head (Cap) size	Handedness	Individually chosen mental task
P01	female	38	56 (56)	right	Visualize horse-racing
P02	female	23	56 (56)	right	Karate movement imagery
P03	male	54	59 (60)	right	Mental planing
P04	male	35	57 (58)	left	Mantra meditation
P05	male	28	58 (58)	right	Mental sex
P06	male	26	56 (56)	right	Mental motorcycling
P07	female	21	56 (56)	right	Mental knitting

Supplementary Table 2. Hyperparameters

Model Variant	Metrics / Estimators	Shrinkages	C values	SVC Metric Options
SuperKernel SVC	rbf, polynomial, laplacian, cosine, poly, corr, cov, scm, lwf, oas, hub, sch, tyl	0, 0.01, 0.1	0.1, 1, 10, 100	euclid, riemann, logeuclid
SuperKernel TLR	rbf, polynomial, laplacian, cosine, poly, corr, cov, scm, lwf, oas, hub, sch, tyl	0, 0.01, 0.1	0.1, 1, 10, 100	–
BlockKernels SVC	rbf, polynomial, laplacian, cosine, poly	0, 0.01, 0.1	0.1, 1, 10, 100	euclid, riemann, logeuclid
BlockKernels TLR	rbf, polynomial, laplacian, cosine, poly	0, 0.01, 0.1	0.1, 1, 10, 100	–
BlockCov SVC	corr, cov, scm, lwf, oas, hub, sch, tyl	0, 0.01, 0.1	0.1, 1, 10, 100	euclid, riemann, logeuclid
BlockCov TLR	corr, cov, scm, lwf, oas, hub, sch, tyl	0, 0.01, 0.1	0.1, 1, 10, 100	–
BlockCovInd SVC	corr, cov, scm, lwf, oas, hub, sch, tyl	0, 0.01, 0.1	0.1, 1, 10, 100	euclid, riemann, logeuclid
BlockCovInd TLR	corr, cov, scm, lwf, oas, hub, sch, tyl	0, 0.01, 0.1	0.1, 1, 10, 100	–
Cov SVC	corr, cov, scm, lwf, oas, hub, sch, tyl	0, 0.01, 0.1	0.1, 1, 10, 100	euclid, riemann, logeuclid
Cov TLR	corr, cov, scm, lwf, oas, hub, sch, tyl	0, 0.01, 0.1	0.1, 1, 10, 100	–
